# Effect of a Very-Low-Calorie Ketogenic Diet on Circulating Myokine Levels Compared with the Effect of Bariatric Surgery or a Low-Calorie Diet in Patients with Obesity

**DOI:** 10.3390/nu11102368

**Published:** 2019-10-04

**Authors:** Ignacio Sajoux, Paula M. Lorenzo, Diego Gomez-Arbelaez, M. Angeles Zulet, Itziar Abete, Ana I. Castro, Javier Baltar, María P. Portillo, Francisco J. Tinahones, J. Alfredo Martinez, Ana B. Crujeiras, Felipe F. Casanueva

**Affiliations:** 1 Department of Medicine, Division of Endocrinology, Complejo Hospitalario Universitario de Santiago (CHUS/SERGAS), Instituto de Investigacion Sanitaria de Santiago (IDIS), 15706 Santiago de Compostela, Spain; ignacio.s@pronokal.com (I.S.); pmarino@alumni.unav.es (P.M.L.); diedgomez@gmail.com (D.G.-A.); anaisabel0102@gmail.com (A.I.C.); 2 Medical Department Pronokal, Protein Supplies SL, 08009 Barcelona, Spain; 3 Faculty of Health Sciences, University of Santander (UDES), Bucaramanga 680003, Colombia; 4 Department of Nutrition, Food Science and Physiology, Centre for Nutrition Research, University of Navarra (UNAV) and IdiSNA, Navarra Institute for Health Research, 31009 Pamplona, Spain; mazulet@unav.es (M.A.Z.); iabetego@unav.es (I.A.); jalfmtz@unav.es (J.A.M.); 5 CIBER de Fisiopatologia de la Obesidad y Nutricion (CIBERobn), Instituto de Salud Carlos III; 28029 Madrid, Spain; mariapuy.portillo@ehu.eus; 6 Division of General Surgery, Complejo Hospitalario Universitario de Santiago (CHUS/SERGAS), 15706 Santiago de Compostela, Spain; javier.baltar.boileve@sergas.es; 7 Department of Nutrition and Food Science, Nutrition and Obesity Group, University of the Basque Country (UPV/EHU) and Lucio Lascaray Research Institute, 01005 Vitoria, Spain; 8 Unidad de Gestión Clínica de Endocrinología y Nutrición, Instituto de Investigación Biomédica de Málaga (IBIMA), Complejo Hospitalario de Málaga (Virgen de la Victoria), Universidad de Málaga, 29010 Málaga, Spain; fjtinahones@hotmail.com; 9 Program for Precision Nutrition, IMDEA, 28049 Madrid, Spain; 10 Laboratory of Epigenomics in Endocrinology and Nutrition, Instituto de Investigacion Sanitaria (IDIS), Complejo Hospitalario Universitario de Santiago (CHUS/SERGAS), 15706 Santiago de Compostela, Spain

**Keywords:** bariatric surgery, body composition, fat free mass, ketogenic diet, obesity, PnK method, protein diet, low-calorie diet, very low-energy diet

## Abstract

The preservation of muscle mass and muscle function after weight loss therapy is currently a considerable challenge in the fight against obesity. Muscle mass secretes proteins called myokines that have relevant functions in the regulation of metabolism and health. This study was aimed to evaluate whether a very low-calorie ketogenic (VLCK) diet may modulate myokine levels, in addition to changes in body composition, compared to a standard, balanced low-calorie (LC) diet or bariatric surgery in patients with obesity. Body composition, ketosis, insulin sensitivity and myokines were evaluated in 79 patients with overweight/obesity after a therapy to lose weight with a VLCK diet, a LC diet or bariatric surgery. The follow-up was 6 months. The weight loss therapies induced changes in myokine levels in association with changes in body composition and biochemical parameters. The effects on circulating myokine levels compared to those at baseline were stronger after the VLCK diet than LC diet or bariatric surgery. Differences reached statistical significance for IL-8, MMP2 and irisin. In conclusion, nutritional interventions or bariatric surgery to lose weight induces changes in circulating myokine levels, being this effect potentially most notable after following a VLCK diet.

## 1. Introduction

Obesity is a worldwide health problem and is considered to be a global epidemic [1,2,3]. Excess body weight is associated with cardiometabolic diseases and certain types of cancer, and weight loss can ameliorate or eliminate the metabolic risk factors related to these morbidities [1,2,3,4]. Diet-induced weight loss is used as treatment for obesity; however, the main problem of these diets is that high amount of fat-free mass—mainly skeletal muscle mass [5]—is also lost together with the fat mass [4,6]; this may increase the risk of sarcopenic obesity [4,6,7]. Sarcopenic obesity imposes a double impact on health because the reduction in muscle mass and muscle strength is also a cause of cardiometabolic disorders [6] and other obesity-related diseases. Weight loss achieved through standard hypocaloric diets occurs at the expense of both fat mass and fat-free mass reduction [4,6,8].

Bariatric surgery results in rapid and massive weight loss (>20% of total body weight) [4] and appears to accelerate the loss of fat-free mass relative to total body or fat mass loss, especially during the first six months after surgery [9].

In contrast, previous studies from our research group have shown that very-low-calorie ketogenic (VLCK) diet is effective for the management of obesity and also for the preservation of muscle mass, because weight loss is achieved primarily via the loss of total fat mass, including visceral fat [6]. This type of diet is characterized by the restriction of carbohydrates and fat intake, with a slight increase in protein intake to the point of inducing a change in metabolism and the generation of plasma ketone bodies.

Recently, the importance of skeletal muscle in weight control has been demonstrated. A cross-talk between skeletal muscle and adipose tissue has been proposed and linked with the control of body weight, both for the fat stores and muscle mass [10]. Skeletal muscle is the largest organ of the body in non-obese individuals [11], accounting for approximately 40% of body weight [11,12] and it is now considered an endocrine organ [11,13]. Similarly, people with normal weight have a higher percentage of muscle mass compared to people with overweight and obesity [14]. Obesity is associated with poor muscle quality, affecting muscle function. This lack in the quality and quantity of muscle mass is directly linked with morbidity and mortality in humans [4].

Skeletal muscle responds to mechanical, neural and humoral stimuli, and plays critical roles in physical activity, energy expenditure, and glucose disposal [11]. The skeletal muscle produces and releases myokines (cytokines and other peptides expressed and released by muscle cells) in response to contraction; these myokines can influence metabolism in other tissues and organs [13]. Myokines are implicated in the autocrine regulation of metabolism in muscles as well as in the paracrine and endocrine regulation of other tissues and organs, including adipose tissue, liver, and brain acting through myokine receptors [12]. Among the most studied myokines thus far are interleukin (IL)-8 [15], IL-6 [11,12,13,16], matrix metalloproteinase-2 (MMP2) [17], and irisin [11,12,16]. IL-6 is the most widely studied exercise-regulated cytokine [13] and is associated with obesity and insulin resistance [13]; but it is also highly produced and secreted after exercise, enhancing insulin action and other metabolic processes [12]. IL-8 is another member of the interleukin family that is a myokine. It is increased in response to exhaustive exercise and potentially involved in angiogenesis within the skeletal muscle [18]. Recently, matrix metalloproteinases, a family of zinc-dependent proteolytic enzymes that belong to a regulatory pathway involved in extracellular remodeling of connective tissues, have attracted interest because of their potential involvement in adipose tissue and muscle function: MMP2 is one of the matrix metalloproteinases [19]. Finally, irisin was proposed as the most relevant mediator of the beneficial effect of exercise, although this is still under debate [12,20].

Therefore, this study was aimed to evaluate whether the VLCK diet modulates the myokine levels, in association with changes in body composition, compared to a standard, balanced hypocaloric diet or bariatric surgery in patients with obesity.

## 2. Materials and Methods

### 2.1. Patients

The study was performed in 79 patients (59 women) with overweight or obesity and 32 normal-weight volunteers (20 women) who served as the control group (Supplementary Table S1). The control group was recruited from companions of patients that agreed to participate in this study; individuals in the control group reported no history of obesity, diabetes mellitus, high blood pressure, or dyslipidemia and were in good health. Patients with obesity underwent a weight reduction therapy based on one of two different energy-restriction programs or bariatric surgery. Thus, a group of patients completed treatment with a VLCK diet (PnK method). The other group was a subsample of the study for the Reduction of the Metabolic Syndrome in Navarra (RESMENA), Spain, a randomized control trial based on a low-calorie (LC) diet. A third group underwent bariatric surgery treatment. Written informed consent to participate in the study was obtained before the start of the study in agreement with the Helsinki Declaration; the study followed national and European Union guidelines. The study was approved by the respective Institutional Ethics Committee for clinical research of Galicia, University of Navarra and Hospital Clinico Virgen de la Victoria from Malaga.

### 2.2. Study Design

#### 2.2.1. VLCK Diet

A cohort of patients subjected to the VLCK diet were enrolled in this study. This cohort included 20 patients with obesity (body mass index (BMI) 35.5 ± 4.4; *n* = 12 women; 47.1 ± 10.2 years) attending the Obesity Unit at the Complejo Hospitalario Universitario de Santiago de Compostela, Spain. The VLCK diet was designed according to a commercial weight-loss program (PnK method), that includes lifestyle and behavioral modification support as described elsewhere [6,21,22,23]. This method is based on high-biological-value protein preparations obtained from cow’s milk, soya, avian eggs, green peas and cereals. Each preparation contained 15 g protein, 4 g carbohydrates, 3 g fat, and 50 mg docosahexaenoic acid, which provided 90 to 100 kcal of energy [24]. The weight loss program had three phases. The first phase consisted of a VLCK diet (600–800 kcal/d) that was low in carbohydrates (<50 g/d from vegetables) and lipids (10 g/d from olive oil) pertaining to 0.8–1.2 g/kg of ideal body weight. Throughout this ketogenic phase, supplements of vitamins and minerals such as K, Na, Mg, Ca, and omega-3 fatty acids were provided. When the target amount of weight was lost, the ketogenic phase ended, and patients started a low-calorie diet (800–1500 kcal/d) followed by a maintenance diet of 1500–2000 kcal/d. The weight loss program had five steps [6] and adhered to the 2015 guidelines of the European Food Safety Authority regarding total carbohydrate intake [25].

Patients followed the different steps of the program for up to a maximum period of 4–6 months, although patients remained under medical supervision for the following 12 months [6,21,22,23]. The intervention included an evaluation by the specialist physician conducting the study and an assessment by an expert dietician. All patients underwent a structured program of physical exercise, with external supervision [24].

Throughout the study, the patients completed a maximum of 10 visits with the research team (every 15 ± 2 days); four visits involved a complete physical, anthropometric, and biochemical assessment, while the remaining visits involved control of adherence to the program and evaluation of potential side effects. The four assessment visits were scheduled according to the development of ketosis for each patient as follows: normal level of ketone bodies (baseline), maximum ketosis (1-2 months), reduction of the ketotic approach because of partial reintroduction of normal nutrition (approximately 3 months), and no ketosis (4–6 months, end of the study) [6]. The total ketosis state lasted for 60–90 days. In all visits, patients received dietary instructions, individual supportive counseling, and encouragement to exercise on a regular basis using a formal exercise program. The compliance of this advice was not registered. The body weight, body composition, and circulating levels of myokines were evaluated at baseline (0 months), at maximum ketosis (2–3 months), and at no ketosis (4–6 months).

#### 2.2.2. Low-Calorie Diet

A group of patients with obesity (*n* = 20, BMI 35.8 ± 4.5; *n* = 10 women; 49.9 ± 9.3 years) followed a therapy program based on a nutritional intervention controlled by trained dieticians from the Department of Nutrition, Food Sciences and Physiology of the University of Navarra, Spain. Briefly, the study lasted six months in two sequential periods; one intervention period of two months (30% energy restriction, i.e., a reduction of 600–800 kcal/d; 40–55% of energy intake from carbohydrates, 30% from lipids and 15–30% from proteins), during which patients received nutritional assessment every 15 days, followed by a self-control period of four months, during which individuals were advised to follow the lifestyle adopted in the first period. The energy-restricted diets prescribed were based on the American Heart Association guidelines and included 3–5 meals per day and a macronutrient distribution of 50–55% of the total caloric value from carbohydrates, 15% from proteins and 30% from lipids. Moreover, all participants were asked to maintain their normal physical activity during the study, although adherence to recommendation was not recorded. Venous blood samples and anthropometric and body composition measurements were collected at baseline (week 0), at the end of the diet intervention (endpoint, 2 months), and 4 months after the end of the treatment (follow-up, 6 months).

#### 2.2.3. Bariatric Surgery

A group of patients with morbid obesity (*n* = 39, BMI 45.6 ± 6.2; *n* = 37 women; 40.8 ± 10.4 years) underwent bariatric surgery by laparoscopic techniques such as Roux-en-Y gastric bypass (*n* = 15, 30.6%), biliopancreatic diversion (*n* = 15, 30.6%), and sleeve gastrectomy (*n* = 19, 38.8%) at the Hospital Clínico Virgen de la Victoria from Malaga and Complejo Hospitalario Universitario de Santiago de Compostela, Spain.

Before surgical intervention, patients were advised to follow a special diet that consisted of liquid diet for one month, soft diet for one month, and subsequently a normal consistency diet providing about 800 kcal/d. Patients subjected to bariatric surgery were provided with a daily vitamin-mineral supplement, beginning on the day of the surgical procedure, to reduce the risk of developing nutritional deficiencies [26]. Patients involved in this study returned to the clinic for all follow-up visits. General recommendations about mobility and exercise were prescribed although adherence to recommendations was not recorded. The follow-up regimen included visits at three and six months at which time anthropometric and body composition measurements were performed, and venous blood samples were collected. Body composition data were available from *n* = 24 patients included in this group.

### 2.3. Anthropometric and Body Composition Measurements

All anthropometric measurements were performed with patients wearing only their underwear and after an overnight fast (8–12 h) according to validated procedures. Body weight and height measurements were performed using a wall-mounted stadiometer (Seca 220 scale, Medical Resources, EPI Inc., Birmingham, AL, USA). The BMI was calculated by dividing body weight by the square of the height (kg/m^2^). Total body composition was measured by dual-energy X-ray absorptiometry (GE Healthcare Lunar, Madison, WI, USA) as described elsewhere [6].

### 2.4. Biochemical Analysis

Venous blood samples were collected after a 12-h overnight fast, and ethylenediaminetetraacetic acid-treated plasma and serum were separated from whole blood and immediately frozen at −80 °C until used.

IL-8 and MMP2 plasma levels were quantified using a commercial multiplex enzyme-linked immunosorbent assay (ELISA) kit (Bio-Plex custom assay, Bio-Rad Laboratories, Marnes-la-Coquette, France) according to the manufacturer’s instructions. These assays showed an inter-assay variability <18.7% and <15%, respectively and an intra-assay precision <7.8% or <10%, respectively.

Plasma levels of IL-6 and irisin were quantified with ELISA (R&D systems and Irisin ELISA Kit EK-067-29, Phoenix Pharmaceuticals Inc., Burlingame, CA, USA, respectively) according to the manufacturer’s instructions. The IL-6 ELISA showed inter-assay variability <6.4% and intra-assay <4.2%. The inter- and intra-assay variability of the irisin ELISA was <15% and <10%, respectively.

Absorbance of each sample was measured in duplicate using a spectrophotometric microplate reader at a wavelength of 450 nm (Versamax Microplate Reader, Associates of Cape Cod Inc., East Falmouth, MA, USA).

Glucose serum concentrations were measured in an autoanalyzer Pentra C-200 (HORIBA ABX, Madrid, Spain) with specific kits. Insulin concentrations were assessed using an ELISA kit available from Mercodia, AB (Uppsala, Sweden) in a Triturus autoanalyzer (Grifols, Barcelona, Spain). Insulin resistance was indirectly determined by the homeostatic model assessment index (HOMA-IR), which was calculated following the formula (fasting plasma glucose (mg/mL) × fasting plasma insulin (mU/L)/405).

Ketosis was determined by measuring ketone bodies, specifically β-hydroxybutyrate (β-OHB), in capillary blood using a portable meter (GlucoMen LX Sensor, A. Menarini Diagnostics, Neuss, Germany) before measurements of anthropometric parameters. As with anthropometric and biochemical assessments, all of the determinations of capillary ketonemia were made after an overnight fast of 8 to 10 h. These measurements were performed daily by each patient during the entire VLCK diet, and the corresponding values were reviewed on the machine’s memory by the research team for managing adherence. Additionally, β-OHB levels were determined at each complete visit by the physician in charge of the patient. In the LC diet and bariatric surgery groups, ketosis was measured in the three points of analysis by the physician; baseline, endpoint and follow-up.

### 2.5. Statistical Analysis

The sample size of the current study was calculated to detect differences in myokine levels considering published values of myokine levels and standard deviations [27,28,29] and it was calculated to achieve an α = 0.05, and a power (1-β) of 80%. The normal distribution of variables was explored using the Kolmogorov-Smirnov and Shapiro-Wilk tests. Analysis of variance (ANOVA) was used to study differences between groups. Repeated-measures ANOVA test was used to study the effects of the time-course of the nutritional therapy program and groupings of body composition, biochemical parameters, and myokine levels in patients with obesity.

Differences with respect to baseline (0 months) were evaluated by Student’s *t*-test within each weight loss treatment. Differences between weight loss treatments were evaluated with univariate ANOVA. Circulating levels of the analyzed myokines presented no statistically significant differences according to gender (data not shown). Therefore, male and female were analyzed together. Moreover, only the concentration of MMP2 was correlated with age (data not shown) and therefore the analysis of differences in MMP2 concentrations was adjusted by age in an analysis of covariance (ANCOVA).

The potential association between body composition and biochemical parameters with myokine levels was evaluated using the Spearman coefficient test. Multivariate linear regression models were fitted to assess the potential association between changes in fat free mass after treatment and adjusted for therapeutic procedures. Three regression models were performed. Model 1 included variables for changes at endpoint from baseline in IL-8, IL-6, MMP2 and irisin. Model 2 included variables for changes at endpoint from baseline in IL-8, IL-6 and MMP2. Model 3 included variables for changes at follow-up from baseline in IL-8, IL-6, MMP2 and irisin.

Statistical analyses were performed for the data from patients who had valid data for all three time-points of the treatment follow-up (1: baseline, 2: endpoint of the treatment, 3: follow-up).

Statistical analyses were performed using SPSS version 22.0 software (SPSS Inc., Chicago, IL, USA) for Windows XP (Microsoft, Redmond, WA, USA). A *p* value ≤ 0.05 was considered statistically significant.

## 3. Results

**Figure 1:**
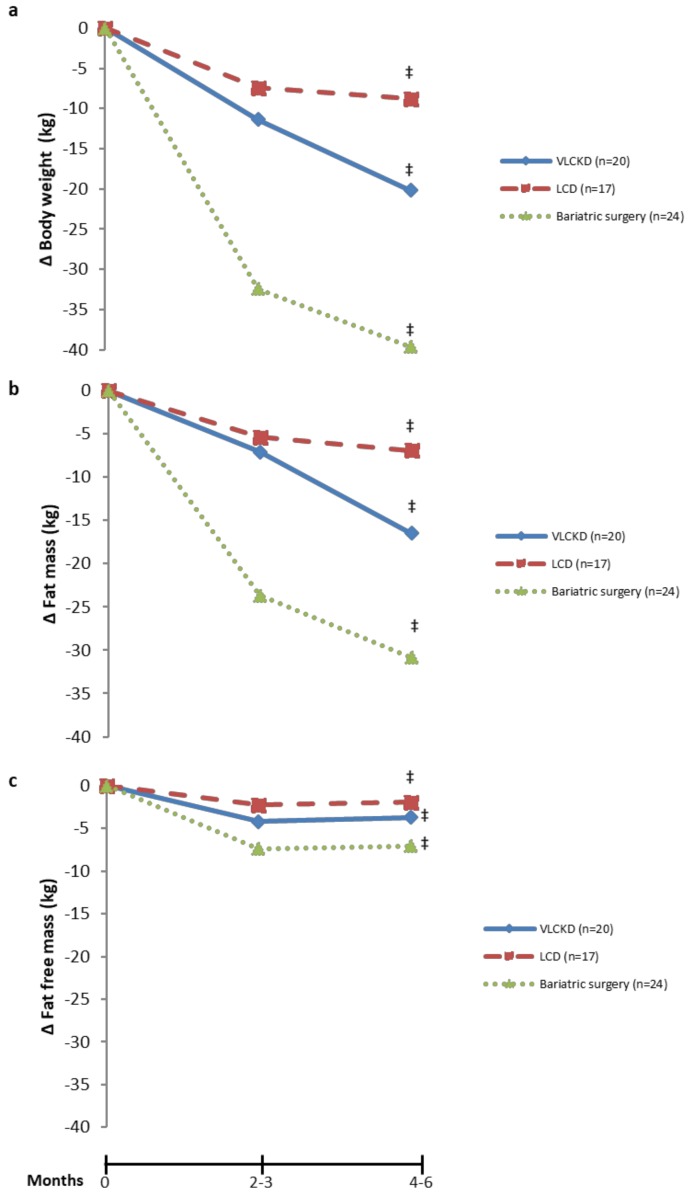
Effect of the weight-loss therapies on body weight and body composition during a follow-up period of 4–6 months. Data show differences compared to baseline for body weight (**a**), fat mass (**b**) and fat-free mass (**c**) after a very-low calorie ketogenic diet (VLCKD), a low-calorie diet (LCD) or bariatric surgery. Data are shown as mean values. ‡ Statistically significant (*p* < 0.05) changes across time calculated with repeated-measures ANOVA.

All patients included in this study reached a statistically significant weight loss (Figure 1a, Table 1). The VLCK diet induced a 20 kg reduction of total body weight, compared with 38 kg induced by the bariatric surgery and 9 kg after the LC diet. This loss in total body weight induced after weight loss therapies was due to both, a loss of fat mass (Figure 1b) and fat-free mass (Figure 1c).

The strong body weight loss induced by the VLCK diet was characterized by a loss of fat mass (16 kg; 39% respect to baseline) but with only a slight loss of fat-free mass; 4 kg (7% with respect to baseline). Patients with obesity showed a 31 kg reduction of fat mass (49.4% with respect to baseline) after bariatric surgery together with a 7 kg reduction of fat-free mass (12.5% with respect to baseline The LC diet induced a 7 kg reduction of fat mass (11.3% respect to baseline) and 2 kg of fat-free mass (Figure 1). The β-OHB levels also differed between weight loss therapies (Table 1). As expected by design, the VLCK diet induced an increase in β-OHB levels, whereas no ketosis was observed after the LC diet. On the other hand, bariatric surgery also induced a mild but statistically significant increase in β-OHB levels. In addition, the three therapeutic approaches induced a significant improvement of HOMA-IR with greatest decrease induced by the VLCK diet (Table 1).

**Figure 2:**
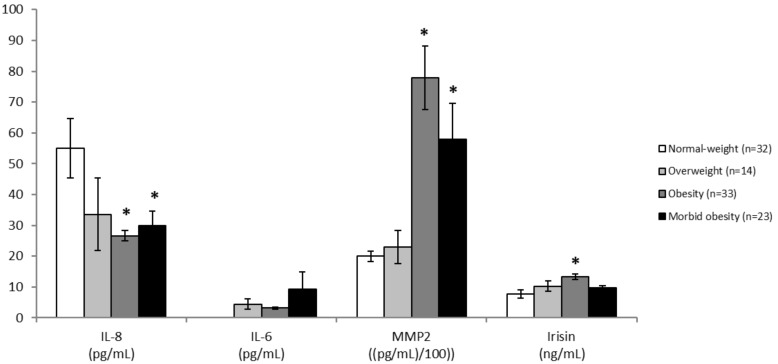
Comparison of circulating levels of myokines according to adiposity at baseline. Differences in interleukin (IL)-8, IL-6, matrix metalloproteinase-2 (MMP2) and irisin levels between normal-weight healthy individuals and patients with excess body weight, classified according to the body mass index as overweight (25–29.9), obesity (30–39.9) and morbid obesity (≥40). Data are presented as the mean; error bars represent the standard error. Asterisk (*) denotes statistically significant differences (*p* < 0.05) in relation to normal-weight individuals evaluated using ANOVA or ANCOVA adjusted for age, as applicable.

The basal levels of myokines IL-8, IL-6, MMP2, and irisin were first evaluated relative to excess adiposity. Thus, the cohort of excess body weight patients were classified according to their BMI as overweight (25–29.9), obesity (30–39.9) and morbid obesity (≥40) and compared with a group of normal weight (BMI < 25) individuals (Figure 2). Circulating IL-8 concentration was lower in patients with obesity than in normal weight individuals and these levels were proportional to the BMI. In contrast, plasma levels of MMP2 and irisin were higher in individuals with obesity than those with normal weight. IL-6 levels in normal weight patients were not measured and no statistically significant differences were observed in patients with obesity according to the BMI classification (Figure 2).

**Figure 3:**
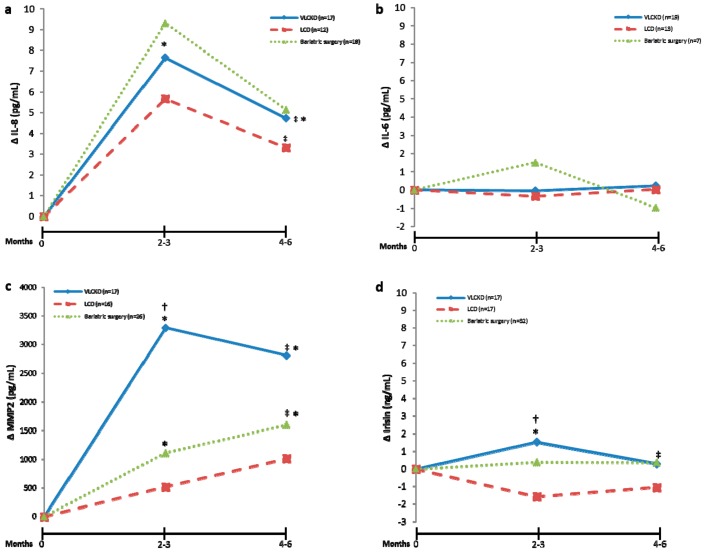
Changes in circulating myokine levels during a very-low calorie ketogenic diet (VLCKD), a low-calorie diet (LCD) or bariatric surgery. (**a**) Changes compared to baseline in interleukin (IL)-8 during the weight loss treatments. (**b**) Changes compared to baseline in circulating IL-6 levels during the weight loss treatments. (**c**) Changes compared to baseline in circulating metalloproteinase-2 (MMP2) levels during the weight loss treatments. (**d**) Changes compared to baseline in circulating irisin levels during the weight loss treatments. Data show differences compared to baseline during the time-course of the intervention. ^‡^ Statistically significant differences over the duration of the nutritional program (from 0 to 4–6 months) evaluated with repeated-measures ANOVA or ANCOVA adjusted for age, as applicable. * Statistically significant differences in relation to baseline evaluated by Student’s *t*-test within each weight loss treatment. ^†^ Statistically significant differences among the three therapeutic procedures to lose weight evaluated by univariate ANOVA.

Following the three therapeutic procedures (Table 1, Figure 3), circulating IL-8 levels increased after the first phase of weight loss treatments, with a tendency to drift back to baseline levels at the end of the study. Notably, this increase was statistically significant for the VLCK and LC diets, but no statistical significance was observed after bariatric surgery (Figure 3a). Considering IL-6, no statistically significant changes were observed after any of the treatment methods (Figure 3b). In addition, MMP2 levels showed an increase that was statistically significant only in patients that followed the VLCK diet or underwent bariatric surgery (Figure 3c). The increase in MMP2 induced by the VLCK diet was particularly evident at maximum ketosis. However, the LC diet did not induce statistically significant changes in MMP2 levels.

Circulating irisin levels increased significantly after the VLCK diet at maximum ketosis and returned to baseline levels at the end of the intervention. In contrast, after bariatric surgery or the LC diet, no statistically significant changes were detected in irisin levels (Figure 3c).

When the changes induced by the weight loss therapies were compared between the types of intervention, it was observed that, particularly during the first phase of the treatments (Figure 3, Table 1; 2–3 months), the VLCK diet could induce larger changes compared to baseline than the changes with bariatric surgery and LC especially for MMP2 (VLCK: Δ3291 ± 5737 pg/mL; bariatric surgery: Δ1039 ± 2798 pg/mL; LC: Δ518 ± 1293 pg/mL, *p* = 0.024) and irisin (VLCK: Δ1.54 ± 2.82 ng/mL; bariatric surgery; Δ0.27 ± 2.94 ng/mL; LC: Δ-1.45 ± 3.22 ng/mL, *p* = 0.010).

Finally, to elucidate whether the changes in the myokine levels were associated with changes in body composition and metabolic characteristics, a correlation analysis was performed using data from all patients (Supplementary Table S2). Under this condition, statistically significant correlations were found between baseline levels of the studied myokines and changes in body weight and body composition. Moreover, MMP2 basal levels in particular were related to an increase in β-OHB level during the weight loss treatments.

**Figure 4:**
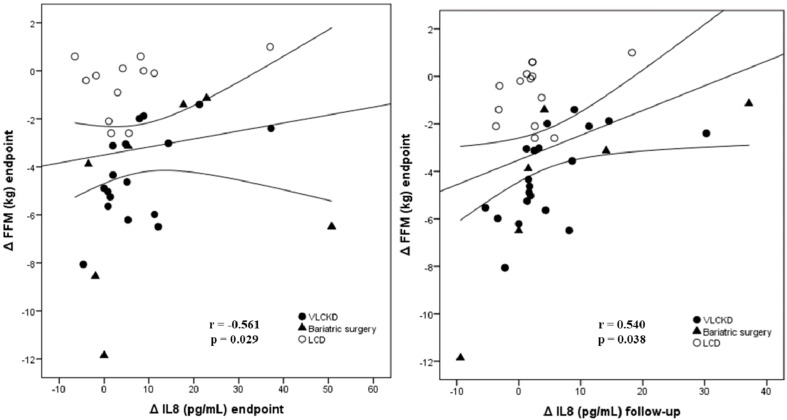
Scatterplot representing the association between weight loss treatment-induced differences from baseline (Δ) at endpoint (2–3 months) in fat-free mass (FFM) and differences in circulating levels of interleukine-8 (IL-8) at endpoint and at follow-up (6 months). In the plots each point refers individual changes. The center line represents the linear regression trendline. The lines above and below the center line represent the upper and lower bounds of the 95% confidence interval around the trendline. VLCKD, very-low calorie ketogenic diet, LCD; low-calorie diet; r, correlation coefficient evaluated by the Rho Spearman test; p, p value.

Considering the effect induced by the weight loss therapies on the studied myokines (Supplementary Table S2), weight loss-induced changes in IL-6 were inversely correlated with changes in fat mass during the first phase of the weight loss treatments. Paradoxically, variations in circulating irisin levels during the first phase of the weight loss treatments were inversely correlated with changes in fat-free mass and also with changes in fat mass at the end of the interventions. When the correlation analysis was adjusted by the intervention group, statistical significance was conserved for the positive association between changes in IL-8 and changes in fat-free mass (Table 2; Figure 4), or HOMA-IR (Table 2) and changes in MMP2 were inversely associated with changes in fat mass (Table 2).

To further assess the effect of the evaluated myokine levels on FFM changes from baseline, linear regression models were performed. After adjustment for therapeutic procedures 54% (*p* = 0.003) of the variability in fat-free mass changes after weight loss treatments, was explained by the combined changes in IL-8, IL-6 and MMP2, especially when changes between baseline and the endpoint were evaluated (Table 3). 

## 4. Discussion

The current study demonstrated that energy-restricted and surgical strategies of weight loss induced changes in levels of circulating myokines such as IL-6, IL-8, MMP2, and irisin in patients with obesity. This effect was more notable in patients following the VLCK diet than the LC diet or bariatric surgery, mainly for irisin and MMP2. The observed changes were related to changes in body composition, especially FFM. In fact, the modifications in circulating myokine levels after weight loss treatments were able to explain more than 50% of the variability in FFM. These results suggest that the concentration in myokines may be a good predictor of changes in FFM after weight loss. As far as we know, this is the first study that evaluated the time-course of myokines after a VLCK diet as compared with a standard LC diet and bariatric surgery. In particular, the results observed in changes in circulating levels of MMP2 support the need for additional longitudinal studies to elucidate the role of this myokine as a protective factor against obesity.

In the last few years, several studies have proposed that skeletal muscle is an endocrine organ and its secreted proteins, termed myokines, are responsible for the improvement of many chronic diseases induced by exercise [16,30]. The preservation of muscle mass and muscle function after a therapy to lose weight is currently a major challenge in the fight against obesity [31]. Most patients with obesity lose fat-free mass or muscle mass after undergoing weight loss therapy [4]. This loss was related to the unsuccessful long-term effect of anti-obesity therapies because the short- or long-term weight regain is mainly promoted by a reduction in the resting metabolic rate. The maintenance of resting metabolic rate is mainly promoted by the maintenance of muscle mass. In this regard, we observed in a previous study that a VLCK diet (PnK method) was able to maintain the skeletal muscle mass and function after four months of nutritional intervention in patients with obesity, who lost an average of 20 kg, with 16 kg of fat mass loss during that time [6]. Accordingly, the resting metabolic rate was preserved following the VLCK diet [32]. Moreover, the changes in fat-free mass after the VLCK diet (PnK method) were slightly lower than those observed after a standard LC diet or bariatric surgery, in accordance with a better metabolic improvement in patients with obesity [33]. Considering these results, this study was aimed to evaluate whether this effect could be reflected on the circulating levels of certain myokines.

Myokines are proteins that were identified in the past few decades, because they are induced after physical activity, suggesting that they could be secreted by muscle cells [13]. In the current study, we observed that the circulating levels of these myokines are dependent on adiposity. The plasma concentration of the studied myokines was different between obesity and normal-weight patients and changed after the weight loss intervention. Accordingly, the magnitude of these changes differed depending on the therapeutic procedure and were more relevant the phase of more active weight loss in the three groups. In this phase patients lost a high amount of body weight, although it was not the maximum of weigh reduction. In the VLCKD group, it was concomitant with the ketogenic phase and in the LC diet it was concomitant with the highest energy restriction followed by a self-control period.

IL-6 is another factor with a dual function, and is related to metabolic disorders [13], however, IL-6 after exercise is associated with metabolic improvements [12]. In this study, we were unable to determine circulating IL-6 levels in patients with normal-weight; thus a comparison between persons with obesity was not possible in this study. In addition, there were not statistically significant differences in IL-6 levels after weight loss interventions. This lack of differences in the current study could be due to the sample size; however, IL-6 is also a relevant proinflammatory cytokine that use to be downregulated after a weight loss treatment [13]. The different patterns and roles of IL-6 in obesity and following exercise may be masking any potential effects, wherein IL-6 is downregulated by weight loss but upregulated by skeletal muscle contractile activity. Previous reports have shown a downregulation of IL-8 gene expression in the adipose tissue [34] and peripheral blood mononuclear cells in humans with obesity treated with a hypocaloric diet; this downregulation is correlated with the loss of fat mass [35]. Therefore, it should be expected that after a VLCK diet (16 kg of fat mass lost) or bariatric surgery (31 kg of fat mass lost), circulating IL-8 levels would decrease. In contrast, in the current study plasma IL-8 levels were lower in obese than in normal-weight individuals and it was associated with a lower FFM. A relevant increase in IL-8 level was observed from the most active phase of weight loss treatment to the follow-up, especially after the VLCK diet. Considering that IL-8 was proposed to be involved in the regeneration of muscle, the increase in IL-8 observed in patients who followed the VLCK diet may be produced by muscles during the VLCK diet and it could be related to muscle mass preservation.

MMP2 was found to be produced and secreted by human adipose tissue and, together with MMP9, was hypothesized to be the key regulator of adipocyte differentiation [36,37]. In accordance with the current study, high levels of MMP2 were previously observed in individuals with obesity. Moreover, serum MMP2 concentration was significantly reduced following bariatric surgery [38], whereas in another study serum MMP2 concentration did not differ before and one year after bariatric surgery [39]. On the other hand, resistance exercise training increased MMP2 levels in both animal models and humans [19]. This exercise-induced increase in MMP2 levels was speculated to be a protective mechanism against obesity and associated diseases such as type 2 diabetes mellitus [40]. In the current work, we observed an increase in circulating MMP2 levels after the VLCK diet, while no statistically significant changes were observed in patients after the LC diet or bariatric surgery. Concordantly, in the current study an increase in irisin was observed after the VLCK diet while it tended to decrease in the LC diet and did not change after bariatric surgery. Irisin was identified as a muscle-derived factor that is secreted after exercise stimulation and was associated with an improvement in metabolic diseases [41]. However, circulating irisin levels were found to be higher in obesity and metabolic syndrome patients compared to normal-weight individuals [20]. Moreover, after weight loss induced by bariatric surgery or calorie restriction, circulating irisin levels decreased [28,42]. In those studies, irisin levels correlated with body weight and fat mass reduction after an intervention of eight weeks on a hypocaloric diet in patients with obesity and the levels returned to baseline after a follow-up period only in patients who regained the lost weight [28]. Considering that no evident changes in fat-free mass were observed in these patients, and that irisin is also an adipokine [43], fat mass was considered the main factor explaining the increase in circulating irisin levels [20]. In contrast, in the current work an increase in irisin levels observed after the VLCK diet occurred despite the marked decrease in fat mass.

Taken together, the increase in myokine levels observed in the current work, particularly following the VLCK diet (PnK method) could be interpreted as a beneficial effect of this ketogenic diet on body composition and metabolic parameters, also found in previous publications [6,32,33,44,45,46]. The longitudinal design of this work is a relevant strength that allows for the evaluation of the time course of changes in circulating myokine levels in association with changes in body weight, body composition, and metabolic parameters within a specific weight loss therapy. Moreover, the relationship between changes in myokine levels and changes in fat-free mass induced by the weight loss therapies, independent of the type of strategy, suggests the suitability of measuring circulating myokine levels as a potential useful tool for monitoring changes in fat-free mass induced by a weight loss therapy.

The most notable difference among the three weight loss treatments in the current study is the instruction of physical activity, dietary-protein enrichment and the induction of ketosis designed in the program of the VLCK diet. Moreover, it is well-known that bariatric surgery induces the loss of muscle mass and fat-free mass that could lead to malnutrition post-surgery because of the occurrence of oral protein intolerance and obesity associated proteinuria. However, although participants following the VLCK diet were instructed to exercise regularly using a formal exercise program, and an increase in physical activity was previously tested during this type of intervention [47], we could not verify adherence to this instruction. Moreover, although there was a slight increase in the loss of fat free mass in patients who underwent bariatric surgery, the differences with the patients who followed the VLCK diet did not reach statistical significance. In addition, biomarkers of protein intake, muscle mass or muscle strength were not evaluated for the three groups and therefore data could not be compared. These are limitations of this study that preclude determining whether changes in physical activity patterns, protein intake or preservation of fat-free mass affected study outcomes. Another differential factor between the VLCK diet and the other therapeutic weight loss approached evaluated in this study is the induced ketosis. This is the first study that evaluated the association between nutritional ketosis and circulating myokine levels. Ketone bodies may also be involved in the regulation of the myokine secretion. In this study, particularly circulating MMP2 levels were associated with levels of β-OHB; this supports the need to perform further studies to explore this topic. 

## 5. Conclusions

**A** VLCK diet for weight loss induced an increase in circulating levels of a representative number of myokines in association with changes in total body weight, fat mass and fat-free mass reduction. Therefore, these results reinforce the suitability of this kind of nutritional treatment in counteracting excess adiposity and its metabolic consequences. In addition, IL-8 circulating levels were modulated after a LC diet and MMP2 levels changed after bariatric surgery. There is a need for further studies to explore the variability of the myokines response to weight loss, with consideration of the complex molecular biology of weight loss, the importance of physical activity to prevent FFM loss after a large weight loss and the potential effect of nutritional ketosis on myokines secretion.

## Figures and Tables

**Table 1:** Changes induced by the weight loss treatments in body weight, body composition and biochemical parameters.

Variable	VLCKD			LCD			Bariatric Surgery			ANOVA *p*-Value		
	Baseline (0 Months)	Endpoint (2–3 Months)	Follow-up (4–6 Months)	Baseline (0 Months)	Endpoint (2–3 Months)	Follow-up (4–6 Months)	Baseline (0 Months)	Endpoint (2–3 Months)	Follow-up (4–6 Months)	Time	Study	Time × Study
Body weight (kg)	96.0 ± 16.3	84.2 ± 13.0 ^‡^	76.6 ± 11.1 ^‡,†,^*	93.0 ± 13.2	89.0 ± 12.5 ^‡^	87.6 ± 12.3 ^‡,†,^*	121.3 ± 21.5	88.9 ± 13.4 ^‡^	81.7 ± 14.3 ^‡,†,^*	<0.001	<0.001	<0.001
Fat mass (kg)	42.2 ± 9.2	35.0 ± 7.8 ^‡^	25.7 ± 5.8 ^‡,†,^*	34.6 ± 8.3	31.4 ± 7.6 ^‡^	30.7 ± 7.6 ^‡,†,^*	62.57 ± 14.9	38.9 ± 7.1 ^‡^	31.7 ± 8.2 ^‡,†,^*	<0.001	0.012	<0.001
Fat free mass (kg)	52.8 ± 10.3	48.6 ± 9.3 ^‡^	49.1 ± 9.7 ^‡,^*	58.3 ± 11.7	57.6 ± 11.6 ^‡^	56.9 ± 11.2 ^‡,†,^*	56.7 ± 9.9	49.3 ± 9.6 ^‡^	49.6 ± 8.5 ^‡,^*	<0.001	0.002	<0.001
β-OHB (mmol/L)	0.3 ± 0.02	1.34 ± 0.13 ^‡^	0.24 ± 0.18 ^‡,†,^*	0.39 ± 0.01	0.38 ± 0.02	0.38 ± 0.01	0.45 ± 0.26	0.51 ± 0.29 ^‡^	0.44 ± 0.30	<0.001	0.942	<0.001
HOMA-IR	4.43 ± 2.37	1.53 ± 0.47 ^‡^	1.71 ± 0.57 ^‡,^*	3.28 ± 1.54	1.65 ± 0.93 ^‡^	1.98 ± 2.31 ^‡,^*	5.21 ± 2.86	2.52 ± 1.18 ^‡^	2.10 ± 1.24 ^‡,†,^*	<0.001	0.029	0.055
IL-8 (pg/mL)	27.5 ± 8.7	35.1 ± 12.7 ^‡^	32.2 ± 8.8 ^‡,^*	22.0 ± 7.5	27.7 ± 7.7	25.3 ± 4.4 *	34.2 ± 17.8	44.1 ± 26.6	39.8 ± 20.3	0.001	0.028	0.758
MMP2 (pg/mL)	7782 ± 6637	11074 ± 11542 ^‡^	10590 ± 11878 ^‡,^*	6782 ± 6524	7301 ± 7113	7793 ± 7455	3700 ± 4077	4813 ± 5527 ^‡^	5300 ± 5654 ^‡,^*	0.001	0.072	0.421
IL-6 (pg/mL)	3.96 ± 3.02	3.92 ± 3.24	4.19 ± 6.33	2.62 ± 1.35	2.27 ± 0.83	2.65 ± 1.68	3.26 ± 1.10	4.77 ± 2.52	2.30 ± 0.51	0.796	0.081	0.872
Irisin (ng/mL)	13.4 ± 3.3	14.9 ± 2.6 ^‡^	13.7 ± 3.2 *	11.9 ± 6.6	10.4 ± 4.7	11.4 ± 7.5	10.6 ± 2.8	10.9 ± 2.8	11.3 ± 2.9	0.869	0.009	0.043

Data represent mean ± standard deviation. * Statistically significant (*p* < 0.05) changes across time evaluated by a repeated-measures ANOVA within each weight loss treatment. ‡ Statistically significant (*p* < 0.05) differences respect to baseline (0 months) were evaluated by Student’s *t*-test within each weight loss treatment. † Statistically significant (*p* < 0.05) differences with respect to endpoint (2–3 months) evaluated by Student’s *t*-test within each weight loss treatment. VLCK, very-low calorie ketogenic diet; LCD, low-calorie diet; IL, interleukin; MMP, metalloproteinase; β-OHB, β-hydroxybutyrate.

**Table 2:** Association between changes in myokine levels and body weight, body composition, HOMA-IR, and β-OHB, with respect to baseline following the weight loss treatments, adjusted by types of intervention.

		ΔIL-8_Endpoint	ΔIL-8_Follow-Up	ΔMMP2_Endpoint	ΔMMP2_Follow-Up	ΔIL-6_Endpoint	ΔIL-6_Follow-up	ΔIrisin_Endpoint	ΔIrisin_Follow-Up
ΔBW_ Endpoint	R	0.237	0.18	−0.309	−0.296	0.057	0.326	−0.454	0.281
	*p*-value	0.395	0.522	0.262	0.285	0.841	0.236	0.089	0.31
ΔBW_ Follow-up	R	0.196	0.194	−0.33	−0.333	−0.032	0.128	−0.304	0.224
	*p*-value	0.483	0.488	0.23	0.225	0.909	0.648	0.271	0.423
ΔFM_ Endpoint	R	−0.195	−0.217	**−0.526**	−0.409	−0.104	0.362	−0.286	0.208
	*p*-value	0.486	0.437	**0.044**	0.13	0.713	0.185	0.301	0.457
ΔFM_Follow-up	R	−0.082	−0.052	−0.415	−0.389	−0.03	0.088	−0.161	0.125
	*p*-value	0.772	0.854	0.124	0.152	0.915	0.755	0.566	0.656
ΔFFM_ Endpoint	R	**0.561**	**0.54**	−0.073	−0.172	0.228	0.161	−0.324	0.362
	*p*-value	**0.029**	**0.038**	0.797	0.539	0.413	0.566	0.239	0.185
ΔFFM_ Follow-up	R	0.443	0.414	−0.074	−0.237	0.113	0.039	−0.25	0.3
	*p*-value	0.098	0.125	0.793	0.396	0.688	0.889	0.369	0.278
ΔβOHB_ Endpoint	R	−0.105	0.05	−0.249	−0.084	−0.151	−0.024	−0.103	−0.458
	*p*-value	0.71	0.86	0.371	0.766	0.591	0.933	0.714	0.086
ΔβOHB_ Follow-up	R	0.193	0.228	−0.031	0.075	0.045	0.342	−0.423	−0.305
	*p*-value	0.491	0.414	0.911	0.79	0.873	0.212	0.116	0.269
ΔHOMA-IR_ Endpoint	R	**0.557**	0.502	0.121	0.153	0.195	0.078	−0.438	−0.146
	*p*-value	**0.031**	0.057	0.667	0.586	0.486	0.782	0.103	0.602
ΔHOMA-IR_ Follow-up	R	**0.573**	**0.586**	0.152	0.184	0.247	−0.039	−0.378	−0.295
	*p*-value	**0.026**	**0.022**	0.589	0.511	0.376	0.889	0.165	0.285

Data represent correlation coefficients as evaluated by the Rho Spearman test in differences (Δ) induced by the weight loss treatments from baseline (month 0) to endpoint (month 2–3) or from baseline (month 0) to follow-up (month 4–6). BW, body weight; FM, fat mass; FFM, fat free mass; βOHB, β -hydroxybutyrate; HOMA-IR, homeostatic model assessment of insulin resistance; R, Rho Spearman correlation coefficient. Bold indicates statistically significant correlation.

**Table 3:** Independent effects of changes from baseline in myokine levels on fat free mass following the weight loss treatments.

	Standardized Coefficients β (95% CI)	*P-*Value
Model 1 ΔFFM_endpoint		
ΔIL-8	0.869 (0.09; 0.28)	0.001
ΔIL-6	0.248 (−0.06; 0.25)	0.195
ΔMMP2_	−0.535 (0.00; 0.00)	0.026
ΔIrisin	0.001 (−0.28; 0.28)	0.996
*Corrected R^2^ = 0.507*		0.008
Model 2 ΔFFM_endpoint		
ΔIL-8	0.868 (0.09; 0.27)	0.001
ΔIL-6	0.248 (−0.05; 0.25)	0.171
ΔMMP2	−0.535 (0.00; 0.00)	0.018
*Corrected R^2^ = 0.540*		0.003
Model 3 ΔFFM_follow-up		
ΔIL-8	0.337 (−0.04; 0.18)	0.180
ΔIL-6	0.159 (−0.14; 0.25)	0.575
ΔMMP2_	−0.081 (0.00; 0.00)	0.771
ΔIrisin	0.020 (−0.29; 0.30)	0.947
*Corrected R^2^ = −0.019*		0.494

FFM, fat-free mass; IL, interleukin; MMP, metalloproteinase; Δ endpoint, differences from baseline (month 0) to endpoint (time-point 2, months 2–3); Δ follow-up, differences from baseline to follow-up (time-point 3, month 4–6).

## Supplementary Materials

The following are available online at https://www.mdpi.com/2072-6643/11/10/2368/s1, Table S1: Baseline participant characteristics, Table S2: Association of myokine levels with body composition, HOMA-IR and β-OHB at baseline or following weight loss treatments.
