# Supplementary material for: Effect of a Very-Low-Calorie Ketogenic Diet on Circulating Myokine Levels Compared with the Effect of Bariatric Surgery or a Low-Calorie Diet in Patients with Obesity
Source: Nutrients. 2019 Oct 4;11(10):2368. doi: 10.3390/nu11102368 (PMC6835835; doi:10.3390/nu11102368)
Supplement: Supplementary file 1 [file nutrients-11-02368-s001.pdf]

Supplementary Table S1. Baseline participant characteristics.

	Normal Weight	VLCKD	LCD	Bariatric Surgery
**N**	32	20	20	39
**Gender (male/female)**	12/20	8/12	10/10	2/37
**Age (years)**	35.6 ± 9.7	47.2 ± 10.2	49.9 ± 9.3	40.8 ± 10.4
**Height (m)**	1.69 ± 0.08	1.64 ± 0.09	1.63 ± 0.1	1.63± 0.10
**Body weight (kg)**	64.9 ± 9.3	95.9 ± 16.3	93.0 ± 13.2	121.3 ± 21.61
**BMI**	22.7 ± 1.7	35.5 ± 4.4	34.6 ± 3.5	44.9 ± 10.4
**FM (kg)**	23.9 ± 7.0	42.2 ± 9.2	34.6 ± 8.3	56.7 ± 9.9
**FFM (kg)**	47.2 ± 9.0	50.2 ± 9.9	58.3 ± 11.7	50.7 ± 9.1

Data show the mean±standard deviation. Abbreviations: BMI, body mass index; FM, fat mass; FFM, fat free mass; LCD, low-calorie diet; VLCKD, very-low-calorie-ketogenic diet.

Supplementary Table S2. Association of myokine levels with body composition, HOMA-IR and β-OHB at baseline or following weight loss treatments.

		**Body Weight**			**Fat Mass**			**Fat Free Mass**			**β-OHB**			**HOMA-IR**		
		**Basal**	**Δ_ Endpoint**	**Δ_ Follow-Up**	**Basal**	**Δ_ Endpoint**	**Δ_ Follow-Up**	**Basal**	**Δ_ Endpoint**	**Δ_ Follow-Up**	**Basal**	**Δ_ Endpoint**	**Δ_ Follow-Up**	**Basal**	**Δ_ Endpoint**	**Δ_ Follow-Up**
**IL-8_**	R	0.076	-0.248	**-0.296**	**0.611**	**-0.691**	**-0.657**	-0.165	**-0.752**	-0.264	0.093	-0.233	-0.060	0.051	0.001	0.046
**Basal**	P-value	0.563	0.054	**0.022**	**<0.001**	**<0.001**	**<0.001**	0.279	**<0.001**	0.083	0.552	0.153	0.707	0.713	0.995	0.741
**ΔIL-8_**	R	-0.118	0.033	-0.012	0.128	-0.115	-0.128	-0.237	0.088	-0.042	-0.087	0.039	0.140	0.159	-0.279	-0.210
**endpoint**	P-value	0.391	0.812	0.930	0.429	0.479	0.438	0.142	0.590	0.800	0.607	0.824	0.417	0.276	0.060	0.152
**ΔIL-8_**	R	-0.098	0.005	-0.030	0.153	-0.161	-0.119	**-0.408**	0.093	-0.049	0.043	-0.074	0.085	-0.152	0.132	0.121
**follow-up**	P-value	0.472	0.968	0.828	0.321	0.296	0.441	**0.006**	0.547	0.750	0.794	0.670	0.602	0.291	0.380	0.402
**MMP2_**	R	0.116	-0.279	-0.201	**0.256**	**-0.313**	**-0.280**	-0.048	-0.207	-0.130	-0.100	-0.101	0.181	0.175	-0.108	-0.074
**Basal**	P-value	0.357	0.055	0.153	**0.040**	**0.030**	**0.045**	0.703	0.158	0.358	0.525	0.584	0.299	0.190	0.489	0.615
**ΔMMP2_**	R	-0.093	-0.084	-0.110	0.124	-0.334	-0.136	**-0.328**	0.155	0.154	0.207	-0.213	-0.326	-0.119	0.063	0.050
**endpoint**	P-value	0.535	0.585	0.473	0.405	0.025	0.372	**0.024**	0.308	0.312	0.226	0.259	0.069	0.436	0.694	0.752
**ΔMMP2_**	R	0.040	**0.308**	0.056	-0.068	0.108	0.076	0.177	0.012	0.082	-0.055	0.167	-0.125	-0.063	-0.164	-0.036
**follow-up**	P-value	0.791	**0.044**	0.714	0.655	0.489	0.618	0.241	0.937	0.589	0.756	0.405	0.497	0.686	0.331	0.817
**IL-6_**	R	-0.137	**0.270**	**0.242**	-0.135	**0.304**	0.175	0.087	0.112	0.066	**-0.363**	**0.374**	**0.309**	-0.219	0.061	0.141
**Basal**	P-value	0.260	**0.025**	**0.047**	0.348	**0.032**	0.229	0.550	0.437	0.651	**0.010**	**0.012**	**0.031**	0.076	0.635	0.259
**ΔIL-6_**	R	0.044	-0.077	-0.106	0.126	-0.074	-0.182	0.148	-0.136	-0.137	-0.232	0.205	0.226	0.019	-0.132	-0.116
**endpoint**	P-value	0.735	0.554	0.417	0.422	0.638	0.250	0.342	0.383	0.386	0.129	0.200	0.145	0.885	0.322	0.375
**ΔIL-6_**	R	0.070	-0.118	-0.134	0.068	-0.090	-0.153	0.059	-0.094	-0.109	-0.217	0.091	0.262	0.049	-0.123	-0.147
**follow-up**	P-value	0.586	0.358	0.294	0.646	0.542	0.298	0.693	0.527	0.462	0.142	0.572	0.075	0.704	0.358	0.254
**Irisin_**	R	0.001	0.010	0.036	-0.131	0.085	0.124	**0.300**	-0.097	-0.067	**-0.456**	**0.403**	0.199	0.114	**-0.252**	-0.052
**Basal**	P-value	0.994	0.931	0.760	0.340	0.539	0.371	**0.026**	0.487	0.629	**<0.001**	**0.005**	0.161	0.353	**0.044**	0.674
**ΔIrisin_**	R	-0.105	0.064	0.028	0.123	-0.068	-0.228	**-0.298**	**-0.273**	**-0.296**	-0.169	0.053	0.123	0.228	-0.096	-0.203
**endpoint**	P-value	0.375	0.588	0.818	0.374	0.625	0.100	**0.029**	**0.046**	**0.031**	0.237	0.724	0.396	0.064	0.449	0.103
**ΔIrisin_**	R	0.061	-0.123	-0.128	0.062	**-0.274**	-0.320	-0.238	-0.072	-0.034	0.117	-0.200	0.018	-0.030	0.118	0.029
**follow-up**	P-value	0.609	0.304	0.279	0.659	**0.047**	0.018	0.083	0.608	0.805	0.415	0.188	0.900	0.806	0.358	0.815

Data represent correlation coefficient evaluated by the Rho Spearman test in differences (Δ) induced by the weight loss treatments from baseline (time-point 1, month 0) to endpoint (time-point 2, month 2–3) or from baseline (time-point 1, month 0) to follow-up (time-point 3, month 4–6). βOHB, β-hydroxybutyrate; HOMA-IR, Homeostatic Model Assessment of Insulin Resistance. Bold indicates statistically significant correlatio
